# Delineating Ecological Protection Policies in Qinghai Province, China: A Twenty-Year Spatiotemporal Evolutionary Grain Production Assessment

**DOI:** 10.3390/foods14173028

**Published:** 2025-08-29

**Authors:** Qi Luo, Yexuan Liu, Jinfeng Wu, Junzhi Ye, Lin Zhen

**Affiliations:** 1 China Aero Geophysical Survey and Remote Sensing Center for Natural Resources, Beijing 100083, China; luoq.18b@igsnrr.ac.cn (Q.L.); wujinfeng@mail.cgs.gov.cn (J.W.); 2 School of Land Science and Space Planning, Hebei GEO University, Shijiazhuang 050031, China; liuyexuan0920@igsnrr.ac.cn; 3 State Key Laboratory of Hydroscience and Engineering, Tsinghua University, Beijing 100190, China; 4 Institute of Geographic Sciences and Natural Resources Research, Chinese Academy of Sciences, Beijing 100101, China; yejunzhi22@mails.ucas.ac.cn; 5 School of Resource and Environment, University of Chinese Academy of Sciences, Beijing 100049, China

**Keywords:** foods production, ecological conservation policies, Qinghai Province

## Abstract

Analyzing the status of food production in Qinghai Province and exploring the nexus between its ecological conservation and food supply are of critical significance. This study systematically synthesizes the evolution of ecological protection policies in Qinghai Province from 2000 to 2020 and delineates the spatiotemporal evolutionary patterns of grain production in Qinghai Province and their underpinning driving factors. The key findings are as follows. (1) From 2000 to 2020, the corpus of policies governing ecological governance measures in Qinghai Province exhibited a sustained growth trend, with management-oriented policies predominating. (2) The primary grain and meat-producing regions in Qinghai Province are predominantly clustered in the northeastern part, displaying a gradual intensification of concentration. From 2000 to 2020, grain production showed an upward trajectory in the northern region and a downward trend in the southern region, whereas meat production exhibited an ascending trend in both the northern and western regions. (3) Agricultural production conditions represent the principal drivers of grain and meat production in Qinghai Province. Specifically, two driving factors—common cultivated area and total power of agricultural machinery—have exerted significant positive effects on grain and meat production across over 30 counties. Ecological protection conditions have manifested heterogeneous effects across different regions of Qinghai Province; the normalized difference vegetation index (NDVI) has exerted a negative influence on grain and meat production in the eastern region while exerting a positive influence in the western region.

## 1. Introduction

Food security, a pivotal issue for human civilization and 21st-century development, constitutes one of the core challenges to sustainable societal progress [1,2]. China, sustaining nearly 20% of the global population with less than 9% of the world’s arable land [3], has made its food security a focus of extensive domestic and international concern [4,5,6]. China’s grain production is characterized by marked regional disparities and temporal variability. Spatially, grain production capacity has declined in southeastern provinces while strengthening in northeastern and western provinces, with the latter emerging as new major grain-producing regions [7,8]. The northward shift of the grain production gravity center has intensified production pressure on northern regions, potentially disrupting regional ecological equilibrium and triggering severe environmental issues, including degradation of cultivated land quality [9].

Situated on the Qinghai–Tibet Plateau within the Three-River-Source Region, Qinghai must confront acute food insecurity challenges. Local agro-pastoral systems grapple with persistent low temperatures, drought, and land degradation, exerting pressure on cropland and grassland resources for food provision [10,11]. While China’s grassland ecological compensation policy (implemented in 2011) has enhanced grassland quality [12], measures such as grazing exclusion and grass–livestock balance have constrained grazing areas and livestock-carrying capacity. Accordingly, Qinghai faces the imperative to balance ecological conservation with agro-pastoral development. Previous food security studies have prioritized farmland protection and crop cultivation in agricultural regions, which are critical for supplying diverse staple and non-staple foods through large-scale systems. However, they have largely neglected the role of pastoral regions in food systems. As one of China’s five major pastoral regions, Qinghai’s livestock products constitute vital local food sources [13]. Integrating grassland resources into local production and land use planning could significantly enhance their contribution to food security and sustainable livelihoods.

Although early food security research centered on food production, internationally comparable production datasets remain scarce [14]. Scholars have employed sub-national datasets and global agricultural censuses to analyze crop production across farm scales [15]. In China, dietary transitions toward animal-sourced products, fruits, and vegetables are intensifying demands for land resources. For instance, Chinese residents’ demand for livestock products has increased by 16% to 30%, implying that China will require an additional 300 × 10^4^–1200 × 10^4^ ha of pasture between 2020 and 2050 [16]. Sustainable intensification emerges as the preferred strategy, yet intensification in isolation may be insufficient to meet future demand unless the total environmental footprint of food production is reduced [17].

To summarize, existing food security research has predominantly focused on supply-side dynamics and determinants in traditional agricultural regions, with a paucity of comprehensive assessments of pastoral food supply. Furthermore, most studies utilize coarse-grained data at national or regional scales, with limited granularity at the county level. Given that grain and meat are important components of the diet of residents in Qinghai, this study compiles 2000–2020 grain and meat production data from 44 county-level units to quantitatively study the spatiotemporal patterns and potential determinants of food (grains and meat) supply and analyzes how ecological protection policies affect the spatiotemporal dynamics of food production in this region. The research hypotheses included the following. In Qinghai Province, measures for ecological governance have shown an upward trend. The production of grain and meat is unevenly distributed across the province and may exhibit a clustering tendency. With the passage of time, the spatiotemporal characteristics of food output will undergo changes. A combination of factors, including climate, agricultural production conditions, social and economic conditions, and ecological protection, exerts an influence on food production.

## 2. Materials and Methods

### 2.1. Study Area

Qinghai Province constitutes the confluence of China’s three major natural regions: the Eastern Monsoon Region, the Northwestern Arid Region, and the Qinghai–Tibet Plateau Region. Specifically, northeastern Qinghai is an agricultural zone encompassing the Hehuang Valley and Qinghai Lake Basin, characterized by relatively concentrated arable land and distinctive grain crop varieties. Northwestern Qinghai contains the Qaidam Basin, which features an arid climate and exhibits agropastoral integration. The southern plateau of Qinghai, among China’s five principal pastoral regions, serves as a key livestock production base, where alpine steppes and alpine meadows dominate the natural grassland types.

**Figure 1:**
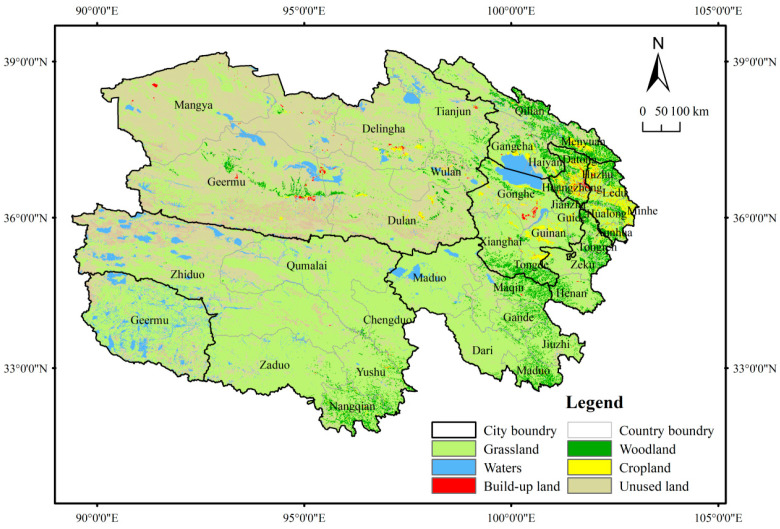
Administrative divisions and land cover of Qinghai Province.

Qinghai administers 8 prefecture-level administrative divisions (2 prefecture-level cities and 6 ethnic autonomous prefectures) and 44 county-level administrative divisions (7 municipal districts, 5 county-level cities, 25 counties, and 7 ethnic autonomous counties) (Figure 1), with a total area of 72 × 10^4^ km^2^. By 2024, Qinghai had a population of 5.93 million, with a population density of merely 8 persons per km^2^, and its population distribution follows a pattern of eastern concentration and western sparsity. The primary sector in Qinghai is dominated by pastoralism, with grasslands covering 56.29% of its total area as of 2020.

### 2.2. Data Collection

#### 2.2.1. Policy Data

A systematic full-text search was conducted using keywords including “ecological protection”, “environmental protection”, “ecological civilization”, “sustainable development”, “grassland”, “forest”, “wetland”, “national park”, “Sanjiangyuan”, and “Qinghai”. The data sources encompassed the State Council Policy Document Database, Peking University Treasure Law Database, the official website of the Qinghai Provincial People’s Government, as well as the websites of Qinghai Provincial Department of Ecology and Environment, the Forestry and Grassland Bureau, the Water Resources Department, and the Agriculture and Rural Affairs Department. These searches aimed to collect textual materials pertaining to Qinghai’s ecological and environmental protection policies. The policy types retrieved include laws, administrative regulations, local regulations, and departmental rules. The issuing authorities include the central government, relevant ministries and commissions, and the Qinghai Provincial Government. The timeframe for policy issuance was 1 January 1998 to 31 December 2020. Following rigorous screening, a total of 95 relevant policy texts were ultimately obtained.

#### 2.2.2. Statistical Survey Data

Two primary food categories consumed by Qinghai residents were selected for this study: grains and meat. Grains, defined herein as food crops, encompass cereals, tubers, and legumes. Meat products include pork, beef, mutton, poultry, and other livestock-derived meats. County-level annual data (2000–2020) on food production, agricultural production conditions, and socioeconomic indicators were sourced from statistical yearbooks and bulletins, including the China County-Level Statistical Yearbook, China Regional Economic Statistical Yearbook, Qinghai Statistical Yearbook, and county-level Statistical Bulletin of National Economic and Social Development for the study period. Variations in statistical standards across years may have compromised the temporal consistency and comparability of the dataset to some extent. To mitigate such impacts on result interpretation, this study collated relevant data from as many sources as possible and performed cross-validation across multiple datasets. The multi-source nature of these data may affect the accuracy of Mann–Kendall tests, especially in counties with incomplete or fluctuating data.

#### 2.2.3. Spatial Data

Remote sensing data on precipitation, temperature, and NDVI (2000–2020) were obtained from the Resource and Environment Science Data Platform. Temperature and precipitation data came from observations at national meteorological stations. They were computed as annual averages via Anuspl spatial interpolation software, with a spatial resolution of 1 km × 1 km. NDVI data were derived from MODIS satellite imagery. They were processed using the maximum-value composite method and resampled to a spatial resolution of 1 km × 1 km. Given that the statistical data used in this study (for example, on food production) were all at county scale, the temperature, precipitation, and NDVI data had to undergo zonal statistics. This calculated county average values, which could then be used for geographically and temporally weighted regression (Section 2.3.5).

### 2.3. Methods

#### 2.3.1. Analysis of Ecological Environment Protection Policies

To examine the evolutionary characteristics of ecological environmental protection policies across different stages, this study employs the word frequency analysis tool within ROST Content Mining System V6.0 to conduct mining on the collected policy texts. Specifically, high-frequency policy terms are first extracted to generate a word frequency statistics file. Subsequently, high-frequency terms irrelevant to policy objectives, measures, subsidies, and the like are manually excluded, and terms with similar meanings are merged to ensure the scientific rigor of the policy text analysis.

#### 2.3.2. Spatial Analysis of Grain and Meat Yield

(1) Standard deviation ellipse analysis and center of gravity migration trajectory model

The standard deviation ellipse analysis and center of gravity migration trajectory model were employed to evaluate the spatial distribution characteristics of major grain- and meat-producing regions and their temporal migration trends. The standard deviation ellipse, rooted in a holistic spatial perspective, is a quantitative geographical method that enables the analysis of centrality and distribution patterns of spatial elements and has been widely used in sociology, epidemiology, ecology, and other fields [18]. The calculation formulas are presented as follows:

$${\bar{X}}_{w}=\frac{\sum_{i=1}^{n}w_{i}x_{i}}{\sum_{i=1}^{n}w_{i}}\;{\bar{Y}}_{w}=\frac{\sum_{i=1}^{n}w_{i}\bar{y}}{\sum_{i=1}^{n}w_{i}}\;A=\sum_{i=1}^{n}{\tilde{x}}_{i}^{2}-\sum_{i=1}^{n}{\tilde{y}}_{i}^{2}\;B=\sqrt{{\left(\sum_{i=1}^{n}{\tilde{x}}_{i}^{2}-\sum_{i=1}^{n}{\tilde{y}}_{i}^{2}\right)}^{2}+4{\left(\sum_{i=1}^{n}{\tilde{x}}_{i}{\tilde{y}}_{i}\right)}^{2}}\;C=2\sum_{i=1}^{n}{\tilde{x}}_{i}{\tilde{y}}_{i}\;tan\alpha =\frac{A+B}{C}\;\sigma_{x}=\sqrt{\frac{\sum_{i=1}^{n}{\left(w_{i}{\tilde{x}}_{i}cos\alpha -w_{i}{\tilde{y}}_{i}sin\alpha \right)}^{2}}{\sum_{i=1}^{n}w_{i}^{2}}}\;\sigma_{y}=\sqrt{\frac{\sum_{i=1}^{n}{\left(w_{i}{\tilde{x}}_{i}sin\alpha -w_{i}{\tilde{y}}_{i}cos\alpha \right)}^{2}}{\sum_{i=1}^{n}w_{i}^{2}}}$$

${(x}_{i}$, $y_{i}$) is the spatial coordinate of the research object, $w_{i}$ represents the corresponding weight; (${\bar{X}}_{w}$, ${\bar{Y}}_{w}$) is the center of gravity of the standard deviation ellipse; α is the azimuth angle; $\sigma_{x}$ and $\sigma_{y}$ are the *x*-axis standard deviation and *y*-axis standard deviation; (${\tilde{x}}_{i}$, ${\tilde{y}}_{i}$) is the coordinate deviation from the location of each research object to the center of gravity.

(2) Local Moran’s Index

The local Moran’s index, a spatial autocorrelation analysis method, was utilized to explore the presence of spatial agglomeration or dispersion in grain and meat yields across the entire province, along with the extent of such agglomeration or dispersion [19]. The calculation formula for this index is as follows:

$$Z_{i}^{*}=\frac{\sum_{j=1}^{n}w_{i,j}x_{j}-\mu \sum_{j=1}^{n}w_{i,j}}{\sqrt{\frac{\left[\begin{array}{c} n\sum_{j=1}^{n}w_{i,j}^{2}-{\left(\sum_{j=1}^{n}w_{i,j}\right)}^{2} \end{array}\right].\sigma^{2}}{n-1}}}$$

$$Local\;Moran\text{’}s\;I=\frac{x_{i}-\mu }{S_{i}^{2}}\sum_{j=1,j\neq i}^{n}w_{i,j}\left(x_{j}-\mu \right)$$

$Z_{i}^{*}$ represents the standardized score of cell i; $x_{i}$ and $x_{j}$ are the grain yield values of the i-th and j-th cell; $w_{i,j}$ is the spatial weights matrix; μ is the average value; σ is the standard deviation. A positive local Moran’s index indicated that grain and meat yield in the i-th county was similar to that of its neighboring counties, that is, high values were surrounded by high values, or low values were surrounded by low values. A negative index indicated that the grain and meat yield in the i-th county was different from that of its neighbors, that is, high values were surrounded by low values, or low values were surrounded by high values.

#### 2.3.3. Temporal Analysis of Grain and Meat Yield

The Mann–Kendall trend test was adopted to analyze temporal trends in grain and meat yields. As a non-parametric test, it does not require data to conform to a specific distribution and is not sensitive to a small number of outliers; thus, it is widely employed in hydrological and meteorological time series analyses.

$$S=\sum_{i=1}^{n-1}\sum_{j=i+1}^{n}sgn(x_{j}{-x}_{i})$$

$$sgn\left(x_{j}{-x}_{i}\right)=\left\{\begin{array}{c} +1\;\mathrm{if}\;x_{j}{-x}_{i}>0\\ 0\;\;\;\mathrm{if}\;x_{j}{-x}_{i}=0\\ -1\;\;\mathrm{if}\;x_{j}{-x}_{i}<0 \end{array}\right.$$

$$Var\left(S\right)=\frac{n\left(n-1\right)\left(2n+5\right)-\sum_{i=1}^{m}t_{i}(t_{i}-1{)(2t}_{i}+5)}{18}$$

One must standardize S to obtain Z:

$$Z=\left\{\begin{array}{c} \frac{S-1}{\sqrt{Var(S)}}\;\;\;\;\;\;\;\;\;\;\mathrm{if}\;S>0\\ \;\;\;\;0\;\;\;\;\;\;\;\;\;\;\;\;\;\;\;\;\;\;\;\;\;\;\;\;\;\;\;\mathrm{if}\;S=0\\ \frac{S+1}{\sqrt{Var(S)}}\;\;\;\;\;\;\;\;\;\;\mathrm{if}\;S<0 \end{array}\right.$$

where S is the sum of the differences between the compared values, and Var(S) is the variance of S. A positive Z value indicates an increasing trend, while a negative Z value indicates a decreasing trend, and the larger the absolute value of Z, the more significant the trend of change in the time series. The significance level *p* < 0.05 corresponds to a confidence level Z_1−α/2_ = 1.96, which means when −1.96 ≤ Z ≤ 1.96, the trend change is not significant; When 1.96 < Z or Z < −1.96, the trend changes significantly.

#### 2.3.4. Screening of Indicators Influencing Food Production

Grain production is shaped by a multitude of factors, including natural conditions, economic development, and social dynamics. Drawing on existing literature and data accessibility, this study incorporates several indicators across four dimensions—climatic conditions, socioeconomic factors, agricultural production conditions, and ecological governance—to construct an indicator system [20,21,22,23]. The climatic dimension includes precipitation and annual temperature, which are the primary climatic factors affecting crop growth. The agricultural production dimension comprises common cultivated area, total sown area of crops and total power of agricultural machinery, which serves as a key indicator influence of crop yield. The socioeconomic dimension encompasses rural population, per capita GDP, disposable income of rural residents, and night-time light intensity, which are employed as commonly used indicators representing human activities and urbanization. The indicator for the ecological governance dimension adopts NDVI, which is an important parameter reflecting the growth status of vegetation and crops. To eliminate the impact of collinearity on these influencing factors, a collinearity test is conducted on the above indicators. The variables are deemed free of collinearity when the variance inflation factor (VIF) is less than 5 and tolerance exceeds 0.1. Indicators exhibiting collinearity (i.e., those whose VIF is more than 5) are excluded. We remove the variables one by one, starting with the highest VIF, and then recalculate iteratively until all remaining variables have VIF values below 5, resulting in a final set of 8 indicators across the 4 dimensions (Table 1).

#### 2.3.5. Geographically and Temporally Weighted Regression

The geographically and temporally weighted regression (GTWR) model integrates temporal attributes and accounts for temporal non-stationarity, building upon the ordinary geographically weighted regression framework [24]. It was employed to quantitatively assess the influence of various factors on grain production, including natural elements (e.g., temperature, precipitation), agricultural factors (e.g., total sown area, cultivated area, mechanization level), economic factors (e.g., per capita GDP, rural per capita disposable income), and anthropogenic factors (e.g., rural population, night-time light intensity). This analysis elucidated the spatial heterogeneity and evolutionary trends of the key driving forces affecting provincial grain production. The model allows for variable estimation of the influence of factors over time and space, thus capturing the regional dynamics of agricultural production. The computational formulas are presented as follows:

$$y_{i}=\beta \left(u_{i},v_{i},t_{i}\right)+\sum_{k=1}^{p}\beta_{k}\left(u_{i},v_{i},t_{i}\right)x_{ik}+\varepsilon_{i}$$

$y_{i}$ and $x_{ik}$ are the observation values corresponding to the independent and dependent variables of the sample point; $u_{i}$, $v_{i}$ are the latitude and longitude coordinates of i sample points; $t_{i}$ represents time; ($u_{i},v_{i},t_{i}$) are the spatiotemporal coordinates of sample points i; $\beta \left(u_{i},v_{i},t_{i}\right)$ is the regression constant for sample points i; $\beta_{k}\left(u_{i},v_{i},t_{i}\right)$ is the k-th regression parameter for sample points i; and $\varepsilon_{i}$ is the residual of the model.

GTWR model parameterization involves two core steps: bandwidth selection and spatial weight matrix construction. Bandwidth selection employs the Akaike information criterion (AIC), with the optimal fixed bandwidth determined via cross-validation to achieve an optimal balance of model fit. The spatial weight matrix is constructed using a Gaussian kernel function, where weights decay exponentially with increasing distance, enabling effective capture of spatial heterogeneity in regional grain production. Model performance is validated using the adjusted R^2^, ensuring the scientific rigor and reliability of parameter settings.

## 3. Results

### 3.1. The Evolution of Ecological Environment Protection Policy

**Figure 2:**
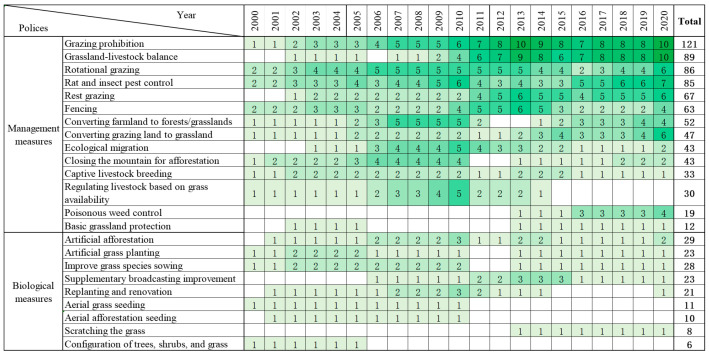
Implementation years of the ecological restoration measures and the number of corresponding policies. Note: The numbers in the color blocks represent the number of policies. If a certain measure appears multiple times in the same policy text, it will be counted as one policy. If different measures appear in the same policy text, they will be counted as different measures.

From 2000 to 2020, 23 major ecological governance measures were implemented in Qinghai Province’s policy practice, comprising 14 management measures and 9 biological measures (Figure 2). Different measures vary in implementation purposes, years, and scopes of application. Management measures are primarily applied to the restoration of degraded grasslands. From 2000 to 2020, the number of management measures adopted in policies increased continuously, with grazing prohibition and grass–livestock balance measures being the most frequently implemented. In 2000, nine measures were introduced in policies for grassland and forest restoration, which included grazing prohibition, rotational grazing, rat and insect pest control, fencing, converting farmland to forests/grasslands, converting grazing land to grassland, closing the mountain for afforestation, captive livestock breeding, and regulating livestock based on grass availability. Since 2002, three additional measures have been implemented: grass–livestock balance, resting grazing, and basic grassland protection and ecological migration. All three are applicable to degraded grasslands. Among them, policies related to grass–livestock balance and rotational grazing have become increasingly common. In contrast, the delineation and protection of basic grasslands are mentioned in only two policy documents. Measures targeting poisonous weed control appeared relatively late in policies, first emerging in 2013.

From 2000 to 2020, biological measures were relatively rarely involved in policies, and they mainly focused on forest and grassland construction. In 2000, three measures were implemented for grassland protection and development: artificial grass planting, improved grass species sowing, and aerial grass seeding. Specifically, artificial grass planting is suitable for grasslands with relatively intact surfaces and gentle slopes, while improved grass species sowing is applicable to degraded grasslands with poor soil quality, and aerial grass seeding is suitable for large-scale pastures. In 2001, three measures for forest protection and construction were added to policies: artificial afforestation, replanting and renovation, and aerial afforestation seeding.

In the early stages, the implementation frequency of management measures and biological measures in policies showed no significant difference. Subsequently, management measures were implemented significantly more frequently than biological measures. This is attributed to the multiple ecological, economic, and social benefits of management measures. For instance, grazing bans and grass–livestock balance measures not only contribute to vegetation restoration but also promote increased production and income for herders. In contrast, most biological measures only exert ecological effects, such as increasing vegetation coverage, conserving water sources, and maintaining soil and water conservation.

### 3.2. Spatiotemporal Patterns of Grain and Meat Yield

#### 3.2.1. Spatial Pattern and Temporal Evolution of Grain Yield

**Figure 3:**
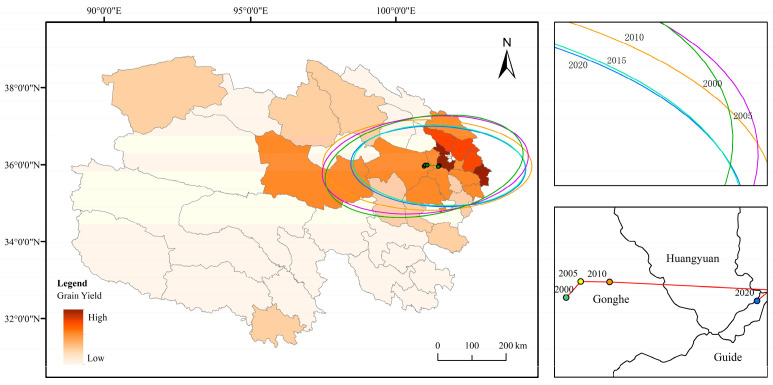
Ellipse of standard deviation and center of gravity migration trajectory of grain production in Qinghai Province from 2000 to 2020.

Grain-producing regions in Qinghai Province are predominantly concentrated in the northeast, with the spatial distribution and developmental trends of total grain output over the past two decades exhibiting distinct directional and clustering characteristics (Figure 3). Between 2000 and 2020, the gravity center of grain production in Qinghai Province exhibited an eastward shift from Gonghe County to Huangzhong County before reverting to Gonghe County, characterized by significant fluctuations in its position, whereas the production center remained relatively stable during other periods. Results from the standard deviation ellipse analysis (Figure 3) indicate that the spatial distribution of total grain production in Qinghai Province from 2000 to 2020 displayed an east–west orientation, with a substantial disparity between the major and minor axes, reflecting a notable flattening rate and distinct directional features. The shorter semi-axis indicates a lower degree of dispersion in the spatial distribution of total grain production over this period, signifying clear clustering. The area of the ellipse showed a downward trend from 2000 to 2020, with a decrease of approximately 30%, suggesting an increase in the concentration of grain production. Over the past two decades, the spatial centripetal force of grain production in Qinghai Province has gradually strengthened, with a more pronounced decline occurring between 2010 and 2015 and more gradual changes in other periods.

All local Moran’s index values for grain production were greater than 0, indicating that spatial autocorrelation was dominated by positive correlations (Table 2). Specifically, counties with high yields tended to form clusters. Grain production in a given county was influenced by that of its neighboring counties, leading to gradual convergence. In particular, counties with high grain yields were clustered in northeastern Qinghai, primarily including Huzhu, Huangzhong, Datong, and Minhe.

**Figure 4:**
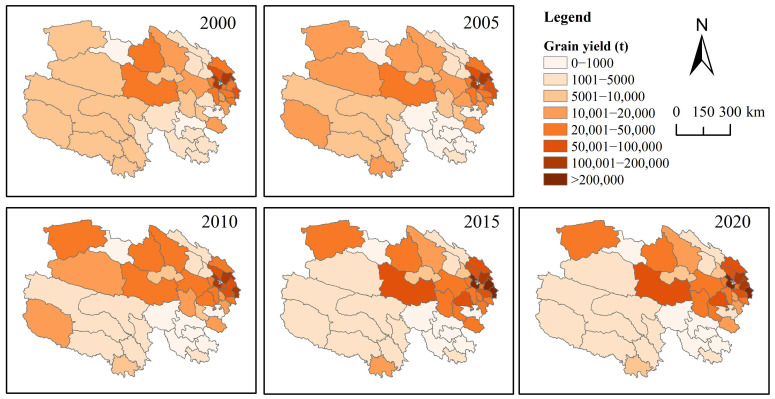
Grain yield from 2000 to 2020 in Qinghai Province.

**Figure 5:**
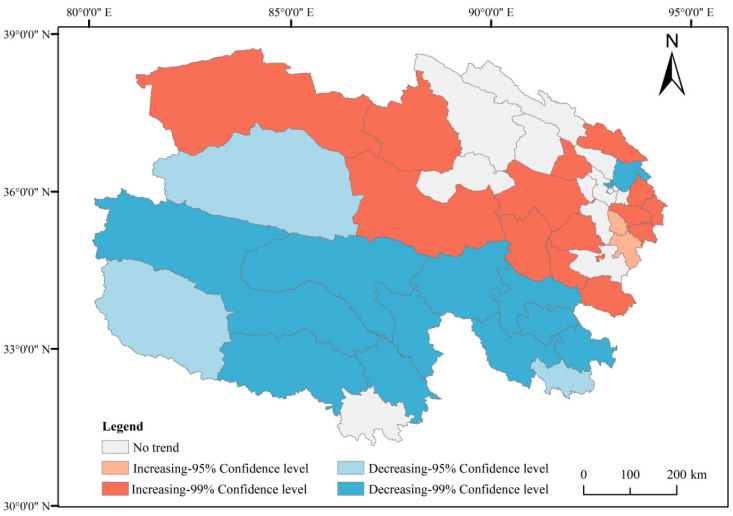
Mann–Kendall test of grain yield from 2000 to 2020.

Trends in grain production changes in Qinghai Province from 2000 to 2020 are illustrated in Figure 4 and Figure 5. Counties exhibiting an upward trend in grain production (95–99% confidence interval) are predominantly concentrated in the northern region, encompassing 16 counties such as Dulan and Gonghe. Counties with a declining trend in grain production (95–99% confidence interval) are mainly distributed in southern Qinghai Province, including 15 counties such as Hujia (note: possible typo, assuming “Huzhu” or another county), most of which are located within the Sanjiangyuan Nature Reserve. Additionally, 13 counties, including Ping’an and Datong, showed no significant trend of change, with these counties concentrated in the northeastern region.

#### 3.2.2. Spatial Pattern and Temporal Evolution of Meat Yield

**Figure 6:**
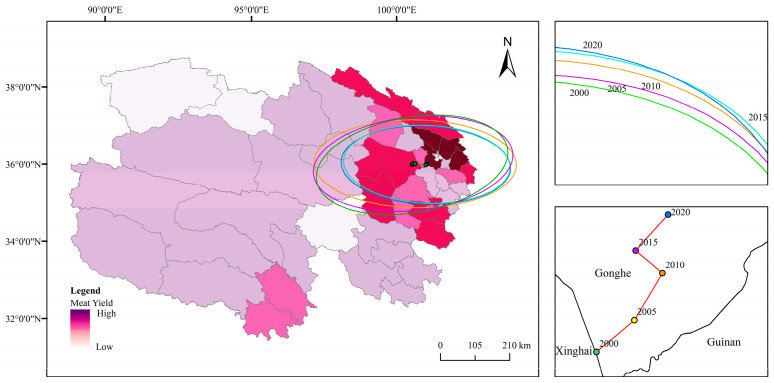
Ellipse of standard deviation and migration trajectory of the center of gravity of meat production in Qinghai Province from 2000 to 2020.

Meat-producing regions in Qinghai Province are predominantly concentrated in the northeast, with the spatial distribution and developmental trends of total output over the past two decades exhibiting distinct directional and clustering features (Figure 6). From 2000 to 2020, the gravity center of meat production in Qinghai Province remained in Gonghe County, with a relatively stable position, indicating a steady development pattern of meat production in the province. Results from the standard deviation ellipse analysis (Figure 6) reveal that the spatial distribution of total meat production in Qinghai Province from 2000 to 2020 exhibited an east–west orientation with a high degree of flatness, reflecting a pronounced directional characteristic. The shorter semi-axis indicates a low degree of dispersion in the spatial distribution of total meat production over the past two decades, signifying clear clustering. The area of the ellipse showed a downward trend from 2000 to 2020, with a decrease of approximately 15%, suggesting an increase in the concentration of meat production. Over the past 20 years, the spatial centripetal force of meat production in Qinghai Province has gradually strengthened, with relatively uniform changes across years.

All local Moran’s index values for meat production were greater than 0, indicating that meat production in a given county was influenced by its neighboring counties, leading to gradual convergence (Table 3). Specifically, counties with high meat yields were clustered in northeastern Qinghai, primarily including Huzhu, Huangzhong, Datong, and Ledu. This phenomenon may be attributed to factors such as grassland resource endowments and policy transmission facilitated by geographical proximity.

**Table 3:** Local Moran’s index of the production for grain and meat at the provincial level in Qinghai.

Food Type	Year	Moran’s I	*Z*-Score	*p*-Value
Meat	2000	0.195	2.409	0.020
	2005	0.182	2.204	0.033
	2010	0.278	3.229	0.002
	2015	0.290	3.441	0.002
	2020	0.344	3.841	0.001

**Figure 7:**
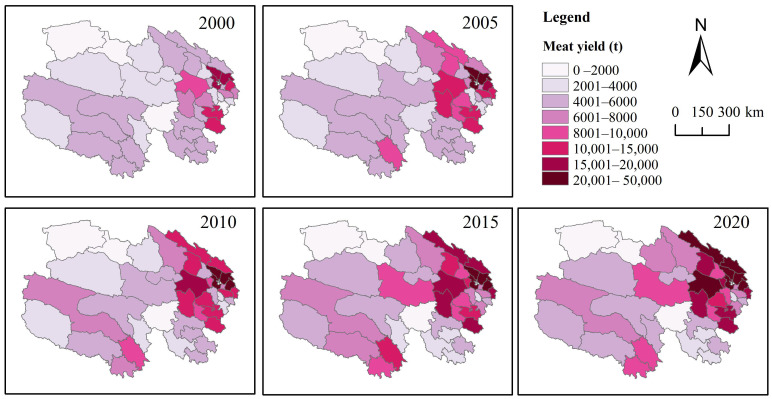
Meat yield from 2000 to 2020 in Qinghai Province.

**Figure 8:**
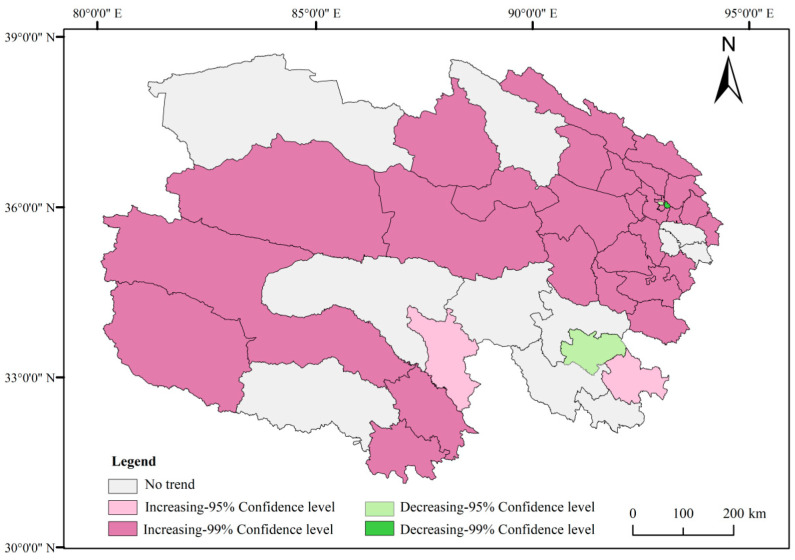
Mann–Kendall test of meat yield from 2000 to 2020 in Qinghai Province.

Trends in meat production in Qinghai Province from 2000 to 2020 are illustrated in Figure 7 and Figure 8. Thirty counties, including Huzhu and Huangzhong, exhibited an upward trend in meat production (95–99% confidence interval), predominantly concentrated in the northern and western regions. Three counties—Chengdong, Chengxi, and Gande—showed a declining trend in production (95–99% confidence interval). Additionally, 11 counties, including Hualong and Xunhua, displayed no significant trend of change.

### 3.3. Factors Controlling Grain and Meat Yield in Qinghai

**Figure 9:**
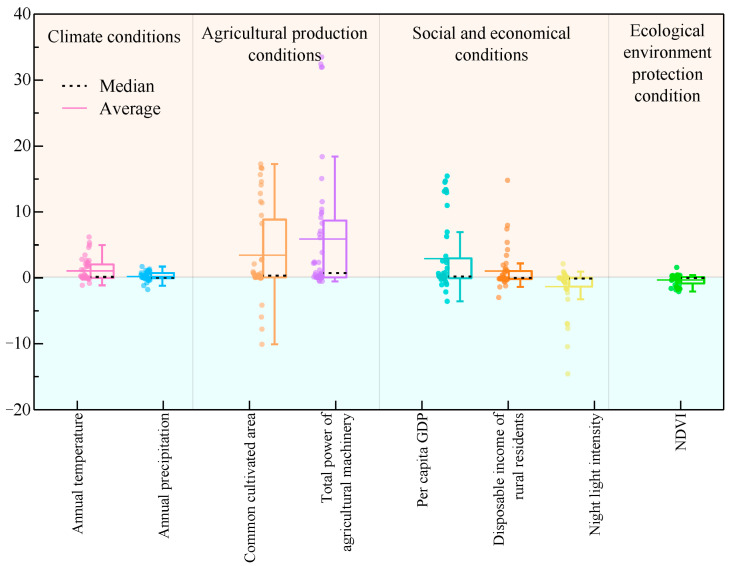
GTWR-based statistical box plot of regression coefficients for grain production determinants in Qinghai Province from 2000 to 2020.

Using the eight selected influencing factors, a GTWR analysis was performed to examine grain yield in Qinghai Province. The model yielded an adjusted R^2^ value of 0.926, indicating a favorable fit. GTWR coefficient results are presented in Table 4 and Figure 9. Overall, agricultural production conditions and socioeconomic factors exert a significant influence on grain yield. The common cultivated area and total power of agricultural machinery both present a positive effect in 36 counties; per capita GDP, disposable income of rural residents, and night-time light intensity show a positive effect in 30, 19, and 14 countries, respectively; NDVI shows a positive effect in 21 countries, and annual precipitation and annual average temperature display a positive effect in 26 and 25 countries, respectively (Table 4). Additionally, the percentage of positive coefficients for annual temperature, annual precipitation, cultivated area, total power of agricultural machinery, per capita GDP, and rural residents’ disposable income exceeds that of negative coefficients. These factors thus exert a positive effect on grain yield, meaning that increases in these variables correspond to increases in grain yield. Conversely, night-time light intensity and NDVI exhibit a negative effect, indicating that increments in these factors would reduce grain production.

**Figure 10:**
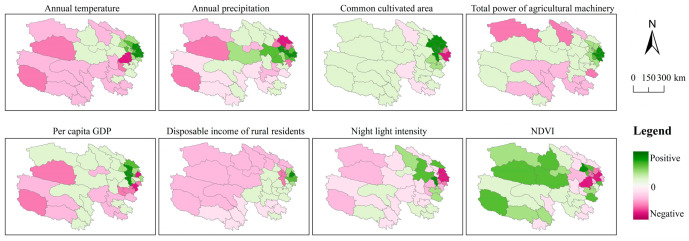
GTWR-based spatial distribution map of regression coefficients for grain production determinants in Qinghai Province from 2000 to 2020.

The spatial distribution of GTWR results for each influencing factor is depicted in Figure 10. With respect to climatic conditions, annual temperature demonstrates a negative effect in 19 counties in the western and southern regions, while exerting a positive effect in 25 eastern counties, with particularly pronounced positive effects in Hualong, Ledu, Minhe, and Huzhu. Furthermore, annual precipitation shows a negative effect in 18 counties in the western and northeastern regions, whereas a positive effect is observed in 26 central counties. Agricultural production conditions constitute the primary positive driver of grain yield. Cultivated area exhibits a positive effect in 36 counties, with marked significance in Huangzhong and Chengbei; total power of agricultural machinery also exerts a notable positive effect, acting as a positive driver in 36 counties (including Hualong and Xunhua) and a negative driver only in 8 counties (including Mangya and Henan). Regarding socioeconomic conditions, per capita GDP shows a positive effect in 30 counties, while rural residents’ disposable income exerts a positive effect in 25 eastern counties. However, night-time light intensity demonstrates a negative effect in 30 counties and a positive effect only in 14 counties (notably, Guide and Gonghe). NDVI exhibits a negative effect in 23 eastern counties (including Pingan and Guinan) and a positive effect in 21 western counties.

**Figure 11:**
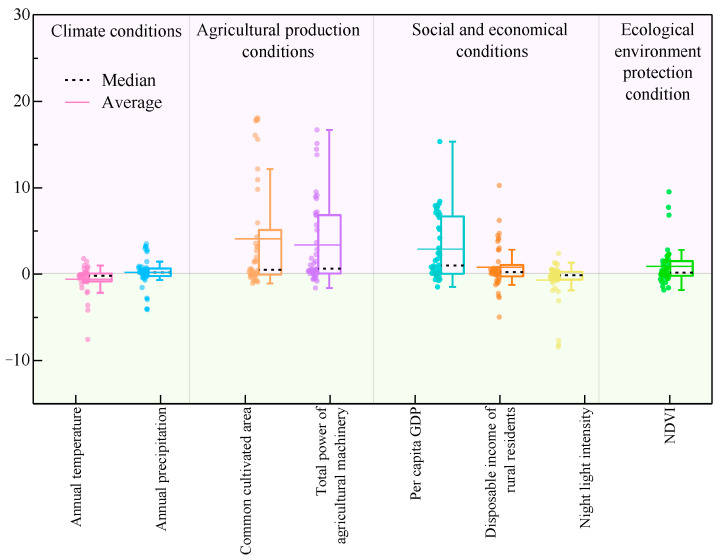
GTWR-based statistical box plot of regression coefficients for meat production determinants in Qinghai Province from 2000 to 2020.

For meat production in Qinghai Province, the GTWR model achieved an adjusted R^2^ value of 0.929, indicating a robust fit. GTWR coefficient results are shown in Figure 11. Overall, agricultural production conditions exert a significant influence on meat yield. Factors such as annual precipitation, cultivated area, total power of agricultural machinery, per capita GDP, rural residents’ disposable income, and NDVI (normalized difference vegetation index) exhibit a higher percentage of positive coefficients relative to negative ones, indicating that these factors exert positive effects on meat production—i.e., meat yield increases as these indicators rise. Common cultivated area and total power of agricultural machinery both present a positive effect in 31 and 35 counties. Per capita GDP, disposable income of rural residents, and night-time light intensity show a positive effect in 35, 30 and 19 countries, respectively; NDVI shows a positive effect in 25 countries; and annual precipitation and annual average temperature display a positive effect in 27 and 13 countries, respectively (Table 4). In contrast, annual temperature and night-time light intensity demonstrate negative effects, meaning that increments in these factors lead to reduced meat production.

**Figure 12:**
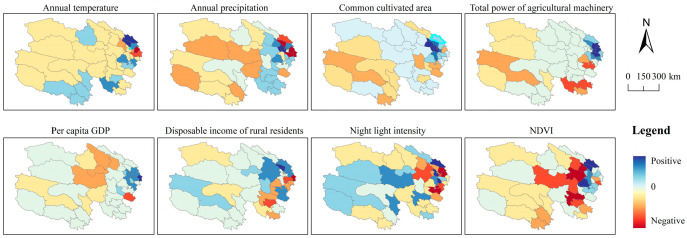
GTWR-based spatial distribution map of regression coefficients for meat production determinants in Qinghai Province from 2000 to 2020.

The spatial distribution of GTWR results for the factors influencing meat production is depicted in Figure 12. Regarding climatic conditions, annual temperature exerts a negative effect on meat production in 30 counties (particularly pronounced in Ledu and Minhe) and a positive effect only in 13 eastern and southern counties. Annual precipitation shows a negative effect in 27 eastern counties and a positive effect in 17 western counties (being notably significant in Chengbei, Huangzhong, and Chengxi). Agricultural production conditions serve as a major positive driver of meat yield: cultivated area demonstrates a positive effect in 31 counties (with marked significance in Huangzhong, Chengbei, Chengxi, and Chengzhong), while total power of agricultural machinery also exerts a notable positive effect, acting as a positive driver in 36 counties (including Ledu and Huzhu) and a negative driver only in 8 counties (e.g., Zeku). With respect to socioeconomic conditions, per capita GDP exhibits a positive effect in 36 counties, and rural residents’ disposable income shows a positive effect in 30 counties. However, night-time light intensity demonstrates a negative effect in 25 counties and a positive effect only in 14 counties (including Menyuan and Huangzhong). NDVI exerts a negative effect in 23 eastern counties (including Pingan and Guinan) and a positive effect in 21 eastern counties.

## 4. Discussion

This study demonstrates that grain production has declined across all counties in southern of Qinghai Province. While meat production increased in a limited number of individual counties within this region, the majority exhibited a downward trajectory. This phenomenon is likely attributable to southern Qinghai’s designation as a priority ecological conservation area, where the primary objective is to mitigate grassland degradation rather than prioritize the development of agriculture and animal husbandry. China established the Three-River-Source National Nature Reserve in 2005 and initiated the pilot program for the Three-River-Source National Park system in 2017, subsequently implementing a series of pivotal ecological protection policies, including the Comprehensive Plan for Ecological Protection and Construction of the Qinghai Three-River-Source Nature Reserve and the Major Project Construction Plan for Ecological Protection and Restoration of the Qinghai–Tibet Plateau Ecological Barrier Zone. During the first phase (2004–2012) of the Three-River-Source ecological protection project, which spans an area of 152,300 km^2^, the trend of grassland degradation in all counties within the project area was effectively contained. However, conservation policies that facilitate vegetation restoration may constrain agricultural expansion in productive regions, thereby indicating a potential trade-off between ecological conservation and agricultural development. From 2000 to 2011, the region experienced an average increase of 11.7% in forage yield and an average decrease of 29.3% in the livestock carrying pressure index [25].

Food supply and demand in Qinghai revealed that grain and meat production during 2012–2020 were predominantly concentrated in the northeastern region [22], which aligns with the spatial pattern of food production observed in this study. We also noted projections indicating a potential increase in future grain demand in northwestern China [26], driven by a dietary transition from staple grains to animal products. This implies a heightened demand for grain feedstock to meet equivalent caloric requirements. Northeastern Qinghai has been identified as a high-risk area for supply–demand imbalances [27]. Therefore, defining the ecological security baseline for Qinghai’s food supply, optimizing intra-regional food supply regulation, and enhancing distribution efficiency are critical for achieving provincial food security [28].

The production of agriculture and animal husbandry are comprehensively influenced by multiple factors. This study indicates that agricultural production conditions and socioeconomic factors exert a significant impact on both grain and meat yields. The examination of the determinants of grain production in Sichuan Province found that agricultural factors dominated the spatiotemporal characteristics of grain output, followed by natural factors [29]. Especially in Daocheng and Jiulong, agricultural production conditions have shown a great positive effect, thanks to the continuous optimization of local agricultural infrastructure. On the one hand, there is a strong development of terraced field improvement and efficient irrigation technology; on the other hand, the local government has made significant investments in infrastructure construction and agricultural technology promotion. This highlights the critical importance of rational allocation of agricultural resources and optimization of natural conditions in ensuring grain production growth, with the preservation of agricultural land area playing a pivotal role in maintaining and enhancing output. Improvements in regional natural resource conditions further augment grain and meat production capacity, which is consistent with the findings of this study.

The impacts of climate change on food production may be long-term and complex; examples include the effects of temperature and precipitation changes on crop growth cycles and livestock breeding. Agricultural production in Qinghai is at high risk of being affected by low temperature and drought. Increasing the construction of irrigation infrastructure and utilizing intelligent facilities to reduce climate disaster risks are urgent requirements in Qinghai. Establishing a predictive model to conduct correlation analysis between long-term meteorological data and production data and revealing how climate changes affect grain and meat production in different regions of Qinghai can provide a scientific basis for regional agricultural and animal husbandry production to cope with climate change.

The impact of population mobility on food production in underdeveloped areas has gradually emerged in recent years. The migration of the population to cities has led to a decrease in the labor force in rural and pastoral areas, affecting the scale and mode of agricultural and pastoral production. It is necessary to analyze the demand changes for food quality, variety, and safety, as well as how these changes feed back into the production process. This provides guidance for agriculture and animal husbandry production to adapt to market demand.

Agricultural and animal husbandry production are also influenced by multiple policies, such as how to achieve synergy between ecological protection policies and agricultural as well as animal husbandry development policies. NDVI exhibits a negative effect in 23 eastern counties and a positive effect in 21 western counties. This uneven pattern may be attributed to the divergent land use dynamics between the two regions. In the eastern counties, where agricultural activities are more intensive, the improvement of vegetation coverage (reflected by higher NDVI) might be associated with the conversion of cropland to ecological land under conservation policies, thereby constraining agricultural production expansion. In contrast, the western counties, with lower agricultural development levels, could benefit from vegetation restoration in terms of enhanced soil fertility and water conservation, which indirectly supports livestock grazing and local agricultural productivity. It is necessary to analyze the effects of these policies in different regions and production processes, comprehensively evaluate the long–term effects and comprehensive impacts of existing policies, and explore paths to optimize policy combinations. In addition, it is also very meaningful to determine changes in the number of livestock raised due to ecological protection policies. It is possible to forecast the scenario of a reduction in the number of livestock, which leads to a decrease in meat production, and to forecast the possible long-term consequences of this trend for food security.

Although this study, through a spatiotemporal analytical framework, clarifies the spatiotemporal dynamic patterns and driving mechanisms of food production in Qinghai, several limitations should be acknowledged. Firstly, constrained by the availability and consistency of county-level statistical data, the research focused exclusively on the spatiotemporal patterns of grain and meat production, without extending to broader food categories such as eggs and dairy products or to more specific food types including wheat, highland barley, potatoes, and oats. Secondly, while the GTWR model effectively elucidates spatiotemporal dynamics, its application is contingent upon data completeness and uniform pixel scale. Future research could explore the applicability of data and methods across larger spatial extents and finer resolutions by integrating multi-modal big data mining.

## 5. Conclusions

From the perspective of spatiotemporal synergistic analysis, this study explores the spatiotemporal evolution patterns of food yield in Qinghai Province and their underlying driving factors. The key findings are summarized as follows.

From 2000 to 2020, the number of policies pertaining to ecological governance measures in Qinghai Province increased steadily, with management-oriented policies dominating, while biological measures remained relatively limited. The grain- and meat-producing regions in Qinghai Province are concentrated primarily in the northeast, and this concentration has increased progressively. From 2000 to 2020, counties with increasing grain yields were mainly distributed in the northern region, while counties with increasing meat yields were distributed in the northern and western regions. This agglomeration will further widen the economic gap between regions, and the concentration of production will drive an increase in infrastructure investment in the northeastern region, while public service resources in underdeveloped areas will be further marginalized, leading to an imbalance in public services. If a reasonable ecological compensation mechanism is lacking for a long time, it will exacerbate the unfairness of the situation where “those who protect suffer losses, while those who develop benefit”. Therefore, in the future, we should optimize the regional functional positioning, strengthen regional synergy, promote resource complementarity, and improve compensation and security policies.

In Qinghai Province, agricultural production conditions stand out as the primary drivers of grain and meat yields, followed by socioeconomic factors. Notably, regarding ecological protection dynamics, the normalized difference vegetation index (NDVI)—a key indicator of vegetation regeneration, often fostered by conservation policies—exerted a negative influence on grain and meat yields in the eastern region while displaying a positive effect in the western region. This divergence underscores a potential tension between environmental protection and food security goals: conservation policies that promote vegetation recovery (reflected in elevated NDVI) may constrain agricultural expansion in productive areas, thereby creating trade-offs between ecological preservation and food production capacity.

We used average temperature and precipitation to study the influence of climate conditions in this research. However, in many cases, maximum and minimum values are more widely used, as certain species or crops are limited by climatic extremes rather than by mean conditions. Thus, research on the impact of extreme climate events on food production is needed in future work.

## Figures and Tables

**Table 1:** Indicators of factors influencing food production.

	Indicators	Units	Tolerance Before Exclusion	VIF Value Before Exclusion	Tolerance After Exclusion	VIF Value After Exclusion
Climate conditions	Annual precipitation	m	0.1997	5.0087	0.2039	4.9035
	Annual temperature	°C	0.6481	1.5429	0.6691	1.4944
Agricultural production conditions	Common cultivated area	ha	0.0314	31.8237	0.2321	4.3076
	Total sown area of crops		0.0287	34.7832	—	—
	Total power of agricultural machinery	Millionsofwatts	0.1752	5.7075	0.2334	4.2837
Social and economic conditions	Rural population		0.1109	9.0186	—	—
	Percapita GDP	CNY	0.8415	1.1884	0.8456	1.1826
	Disposable income of rural residents	CNY	0.6229	1.6054	0.6536	1.5299
	Night-time light intensity	/	0.5547	1.8026	0.5955	1.6793
Ecological environment protection conditions	NDVI	/	0.1923	5.1996	0.2060	4.8548

Note: — indicates that the indicator has been excluded due to severe collinearity and will not be involved in the analysis of influencing factors.

**Table 2:** Local Moran’s index of grain production at the provincial level.

Food Type	Year	Moran’s I	*Z*-Score	*p*-Value
Grain	2000	0.216	2.742	0.017
	2005	0.237	2.992	0.009
	2010	0.267	3.294	0.007
	2015	0.268	3.450	0.004
	2020	0.253	3.350	0.008

**Table 4:** The number of counties with positive and negative influences on each indicator.

		Grain		Meat	
		Positive	Negative	Positive	Negative
Climate conditions	Annual precipitation	26	18	27	17
	Annual average temperature	25	19	13	31
Agricultural production conditions	Common cultivated area	36	8	31	13
	Total power of agricultural machinery	36	8	35	9
Social and economic conditions	Per capita GDP	30	14	35	9
	Disposable income of rural residents	19	25	30	14
	Night-time light intensity	14	30	19	25
Ecological environment protection conditions	NDVI	21	23	25	19

## Data Availability

The original contributions presented in the study are included in the article, further inquiries can be directed to the corresponding author.
